# Traumatic elbow luxation in cats: A cadaveric study on the role of collateral and olecranon ligaments in joint stability

**DOI:** 10.1111/vsu.14314

**Published:** 2025-07-07

**Authors:** Martin Hamon, Pradeep R. Malreddy, Andrew K. Curtis, Pierre P. Picavet

**Affiliations:** ^1^ Department of Clinical Sciences, College of Veterinary Medicine Kansas State University Manhattan Kansas USA; ^2^ Department of Anatomy and Physiology, College of Veterinary Medicine Kansas State University Manhattan Kansas USA

## Abstract

**Objective:**

To determine the role of each part of the collateral ligaments and the olecranon ligament in traumatic elbow luxation pathophysiology in cats.

**Study design:**

Feline cadaveric study.

**Sample population:**

A total of 15 cats and 30 thoracic limbs tested.

**Methods:**

Attempts were made to manually luxate (laterally, medially, and caudally) each elbow after sequential section of each part of the medial collateral ligament, the lateral collateral ligament, and the olecranon ligament by direct and indirect forces applied to the antebrachium.

**Results:**

No elbow luxation occurred with indirectly applied rotational forces. Luxation was only possible with direct forces applied to the antebrachium (axial compression and varus/valgus/caudal forces). Lateral elbow luxation was possible after transection of medial and lateral collateral ligaments or after transection of olecranon and medial collateral ligaments. Medial luxation was possible after transection of olecranon and lateral collateral ligaments. Caudal luxation was possible with transection of olecranon and lateral collateral ligaments.

**Conclusion:**

Various combinations of ruptured ligaments can account for lateral, medial, and caudal luxations of the elbow. The olecranon ligament plays a major role in elbow stability.

**Clinical significance:**

Traumatic elbow luxation in cats can happen with only one collateral ligament rupture, if the olecranon is concurrently severed. The integrity of each ligament should be assessed before any repair.

**Abbreviations**
cdMCL: caudal part of medial collateral ligament
crMCL: cranial part of medial collateral ligament
LCL: lateral collateral ligament
MCL: medial collateral ligament
OL: olecranon ligament

## 1 INTRODUCTION

Traumatic elbow joint luxation is an uncommon condition in cats.^1^ It has been reported to occur following high energy indirect rotational forces rather than direct traumatic forces.^2^, ^3^, ^4^, ^5^, ^6^ Direct forces are thought to be only able to cause a luxation if the elbow is flexed at an angle of 45° or less.^7^, ^8^, ^9^

The stability of the elbow joint is provided by bony conformation, strong collateral ligaments, and the anconeal process that interlocks in the olecranon fossa preventing luxation when the joint is in the extended position.^10^, ^11^, ^12^ Lateral luxation is more common than medial luxation due to the anatomical conformation of distal humerus.^4^ The distal slope and the large size of humeral trochlea prevent medial displacement of the radius and ulna.^1^, ^13^, ^14^, ^15^ Another factor for the overrepresentation of lateral luxation is the difference between the lateral and medial collateral ligaments. The medial collateral ligament (MCL) is weaker and has a slim insertion, while the lateral collateral ligament (LCL) is larger, with a fan‐shaped insertion and more elastic due to the presence of the collagen fiber bundles that cross at varying angles.^3^, ^13^

There is some discrepancy in the collateral ligaments' anatomic description in cats, particularly concerning the insertion of the lateral collateral ligament. Vollmerhaus et al.^16^ and Frewein and Vollmerhaus^17^ reported that, similar to the dog, the collateral ligaments of the elbow in cats consist of two parts each (radial and ulnar parts), but their course and relative strength differ. The medial collateral ligament originates on the medial humeral epicondyle and divides into cranial and caudal crura. The less robust cranial crus inserts on the radius, whereas the stronger caudal crus primarily inserts on ulna, with a partial connection to the radius as well. Laterally, the collateral ligament originates on the lateral humeral epicondyle and divides also into two crura. The cranial crus inserts on the radius and the stronger caudal crus inserts on the ulna (at the level of lateral coronoid process). Engelke et al.^18^ also studied feline elbow anatomy. They found that the lateral collateral ligament (LCL) has a superficial and a deep part, both originating from the humerus. Fiber bundles of the deep part insert into the annular ligament, while the remaining deep fibers and the superficial part insert with a long antebrachial portion on the radius. The medial collateral ligament (MCL) originates from the medial humeral epicondyle and divides into cranial (crMCL) and caudal (cdMCL) parts. The caudal part inserts medioproximally on the ulna, while the cranial part attaches primarily with a long thin part to the caudal aspect of the radius.

Collateral ligaments play a crucial role in elbow stability.^19^ A cadaveric study investigating the pathophysiology of elbow luxation in cats found that lateral elbow luxation could only be induced by transecting both the medial and lateral collateral ligaments.^1^ However, the specific part of the collateral ligaments that were transected were not specified. Additionally, in this study, elbow luxation was observed in only two out of six elbows. Despite this, this finding has been often translated as “In cats, luxation of the elbow joint requires transection of both the medial and lateral collateral ligaments”.^20^

The olecranon ligament (OL) is a structure rarely mentioned and investigated in veterinary medicine.^1^, ^6^, ^18^, ^20^, ^21^, ^22^ This ligament has been described to originate at the lateral surface of the medial humeral epicondyle and inserts at the cranial crest of the olecranon extending distally to the roof of the anconeus process. The wide/large olecranon ligament would help to limit the maximal flexion of the elbow joint and limit lateral movement of the ulna during elbow flexion.^18^, ^21^ However, the role of the olecranon ligament in elbow luxation pathophysiology has never been studied.

The objective of the study was to determine the role of each part of the collateral ligaments and the olecranon ligament in traumatic elbow luxation.

## 2 MATERIALS AND METHODS

### 2.1 Preliminary anatomical evaluation

A preliminary anatomical evaluation was conducted using a single formalin‐preserved feline cadaver (two thoracic limbs) to confirm the anatomical locations of the medial and lateral collateral and olecranon ligaments.

**FIGURE 1:**
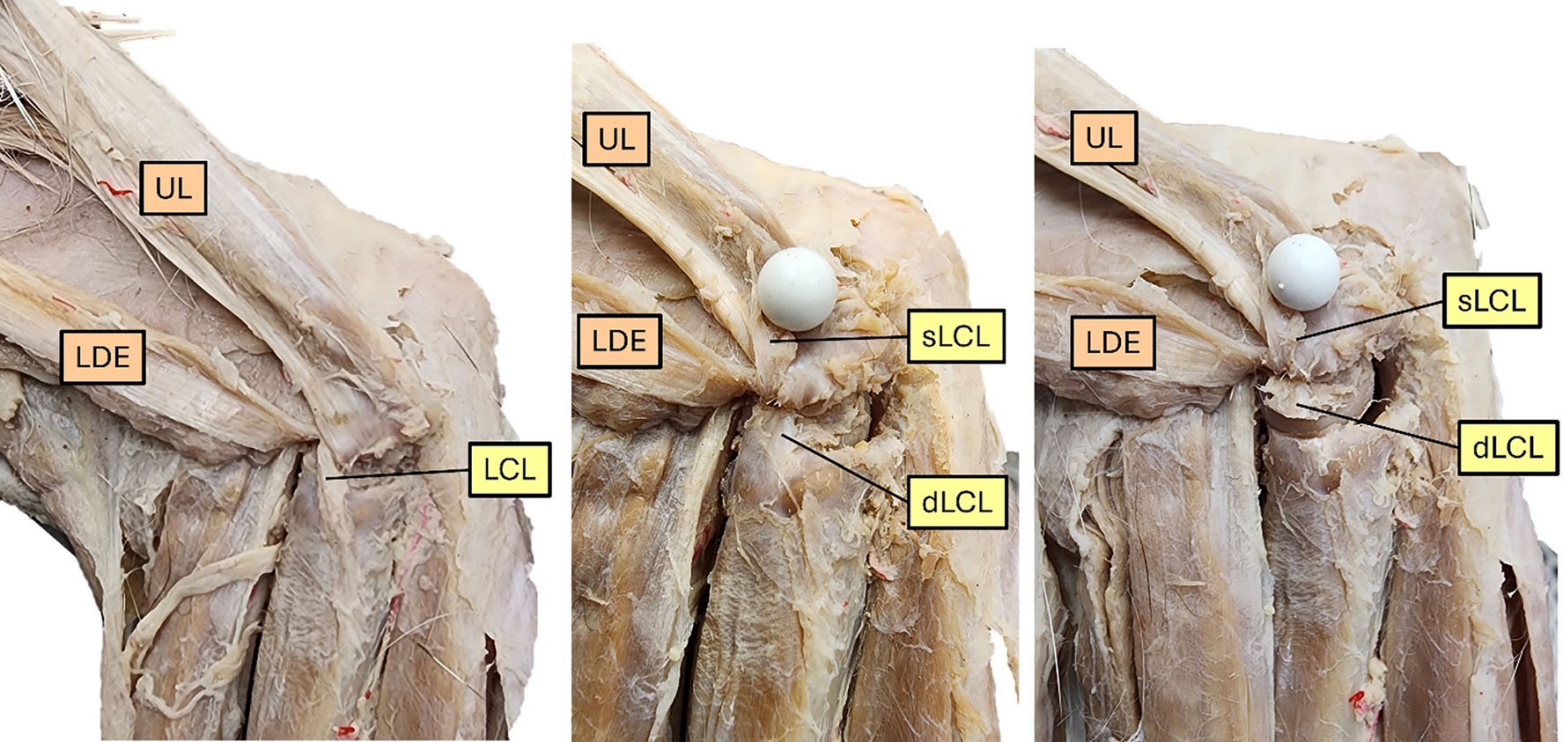
Representative lateral cubital joint region dissected to expose the lateral collateral ligament (LCL) and its superficial (sLCL) and deep (dLCL) parts in a preserved specimen. Other structures shown include the lateral digital extensor (LDE) and ulnaris lateralis (UL).

**FIGURE 2:**
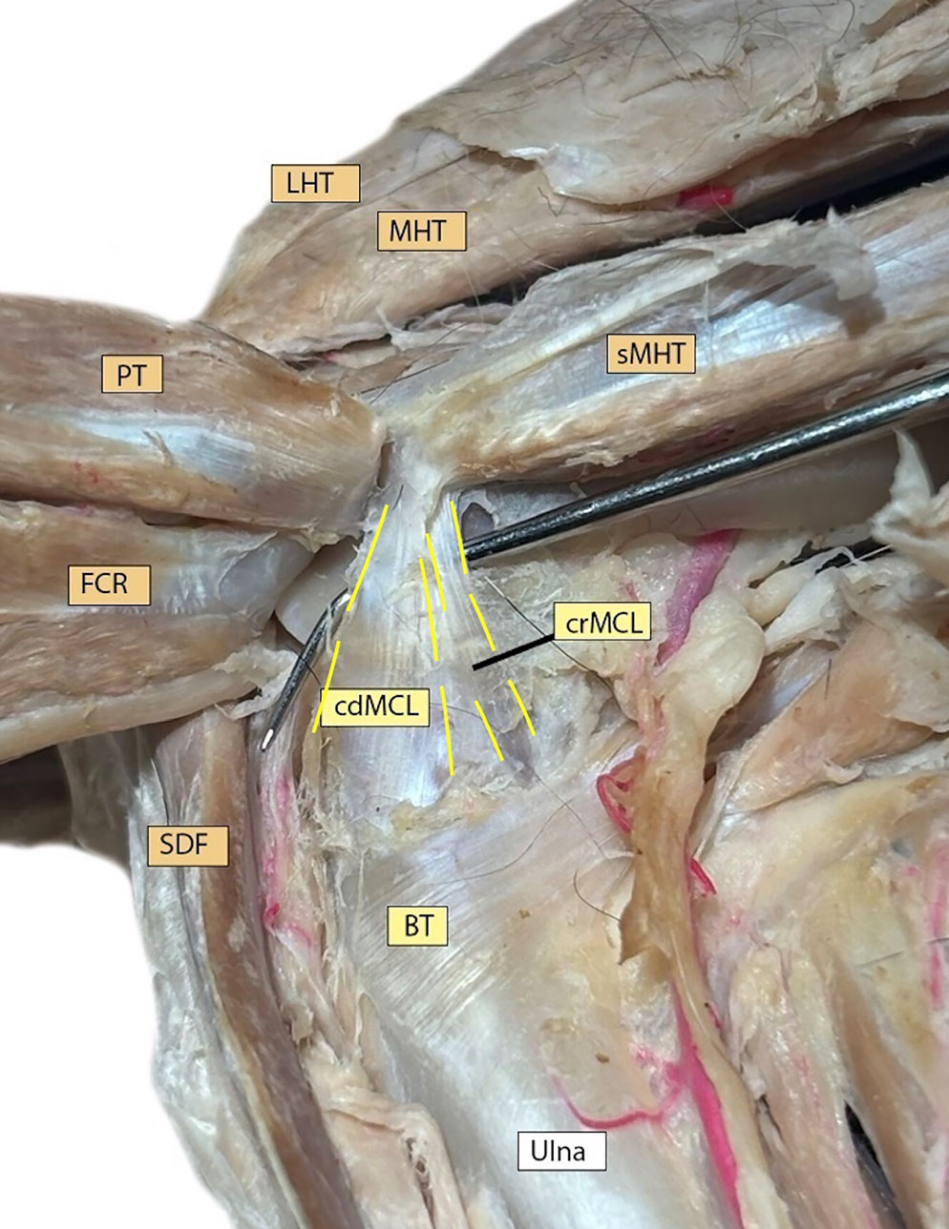
Representative medial cubital joint region dissected to expose the caudal (cdMCL) and cranial (crMCL) portions of the medial collateral ligament (MCL) in a preserved specimen. The probe is located under the medial collateral ligament. Other structures shown include the pronator teres (PT), flexor carpi radialis (FCR), superficial digital flexor (SDF), tendinous insertion of the brachialis (BT), ulna, medial head of triceps (MHT), short part of the medial head of triceps (sMHT), and long head of triceps (LHT).

The lateral and medial collateral ligaments originated proximally from the lateral and medial epicondyles of the humerus, respectively. To expose the lateral collateral ligament (Figure 1), the lateral digital extensor and ulnaris lateralis muscles were identified and traced to their proximal origin on the lateral epicondyle. Similarly, the medial collateral ligament (Figure 2) was exposed by removing the medial head of the triceps brachii muscle and reflecting the brachialis and pronator teres muscle insertions.

The lateral collateral ligament (LCL) was observed inserting exclusively on the radius, with no attachments to the ulna. The ligament consisted of two distinct parts: a superficial and a deep portion. The superficial and longer part attached to the proximal extremity of the radius, whereas the deeper and shorter part blended with the annular ligament, which encircles the head of the radius. No direct ulnar attachments were identified.

The medial collateral ligament (MCL) was observed dividing into a wide caudal part (cdMCL) and thin cranial part (crMCL). The caudal portion attaches medioproximally to the ulna, while the cranial portion connects mainly with a long, thin segment to the caudal side of the radius.

**FIGURE 3:**
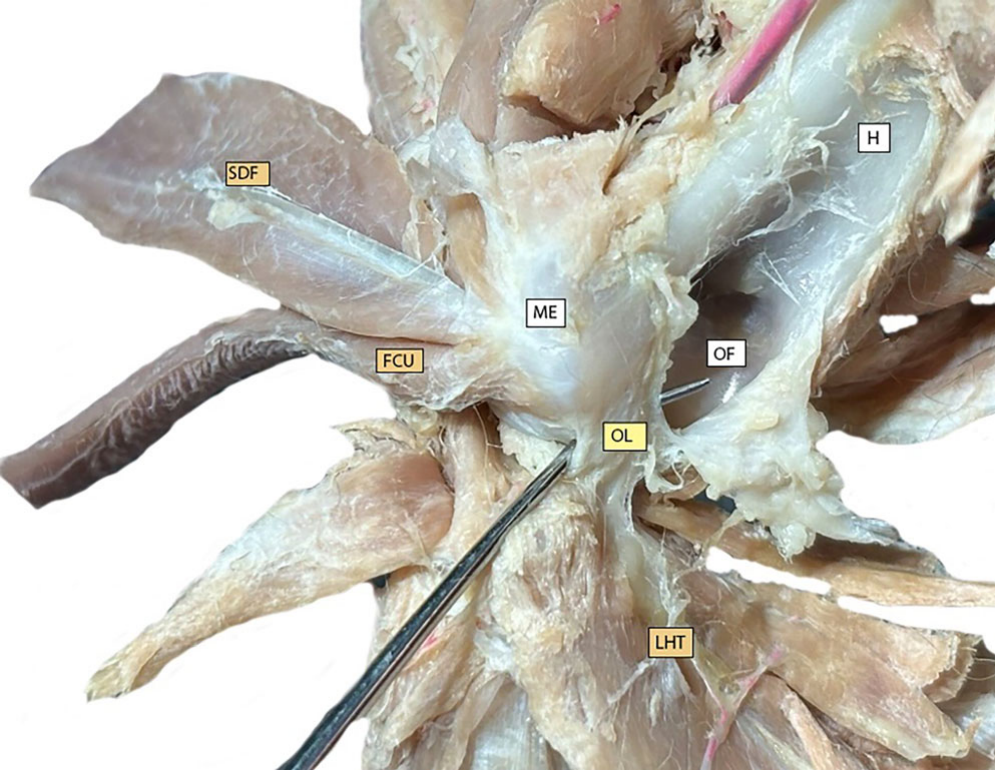
Representative caudomedial cubital joint region dissected to expose the olecranon ligament (OL) in a preserved specimen. The probe is located under the OL. Other structures shown include the superficial digital flexor (SDF), flexor carpi ulnaris (FCU), long head of triceps (LHT), olecranon fossa (OF), humerus (H), and medial epicondyle (ME).

The olecranon ligament was then isolated, running from the lateral surface of the medial humeral epicondyle and inserted on the cranial crest of the olecranon, extending distally to the roof of the anconeal process. (Figure 3).

### 2.2 Specimens and preparation

Specimens were skeletally mature cats (*n* = 15) obtained after humane euthanasia for reasons unrelated to this study (IACUC‐5177). The cadavers were fresh frozen at −21°C. Prior to testing, the cadavers were thawed at room temperature for approximately 24 h. Limbs were then preconditioned before data collection to make sure the mobility of the elbow joint was not compromised. This was accomplished manually by 50 repetitions of passive flexion and extension throughout the full range of motion of the shoulder, elbow, and carpal joints. Skin was left on the specimens.

### 2.3 Specimen testing

Ligaments were sectioned sequentially using a #11 scalpel blade and several tests were performed to induce elbow luxation. All the tests were performed by the same operator (MH). The approach to the elbow joint was the same as previously described.^23^ The superficial and deep parts of the lateral collateral ligament were both transected together.

The study was divided into two parts (I and II). They both aimed at investigating the specific roles of each ligament (LCL, crMCL, cdMCL, OL) in the pathophysiology of elbow luxation.

**FIGURE 4:**
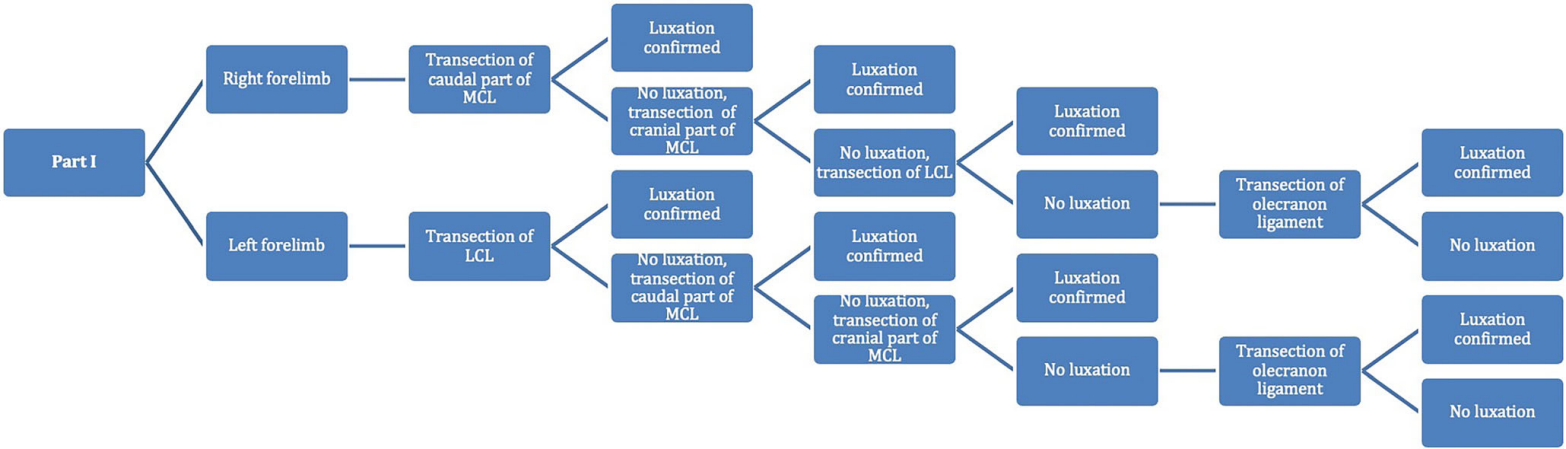
Part I. Sequence to investigate the role of each ligament in the pathophysiology of elbow luxation, starting with collateral ligament transection.

**FIGURE 5:**
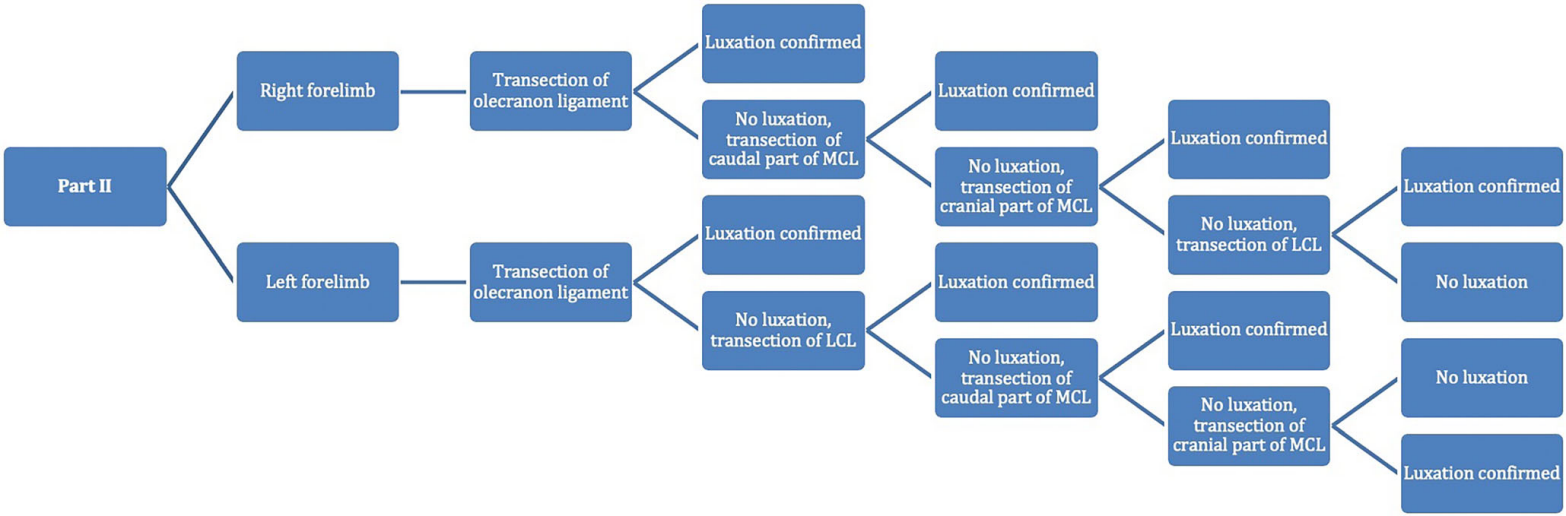
Part II. Sequence to investigate the role of each ligament in the pathophysiology of elbow luxation, starting with olecranon ligament transection.

Figures 4 and 5 show the transection sequences performed. In part I (Figure 4), the sequence starts with collateral ligaments transection, and then olecranon ligament transection whereas in part II (Figure 5), the sequence starts with olecranon ligament transection.

Following each ligament transection (LCL, crMCL, cdMCL, OL), two different tests were performed:

- Attempt to induce luxation of the elbow laterally or medially using indirectly applied rotational forces (manual pronation and supination of the manus). The applied force was the maximum that could be generated with one hand of a single operator (MH) and the full lever‐arm of the manus as described in Farrell et al.^1^ This test is an extreme version of the Campbell test, pushed beyond its limits.
- Attempt to induce luxation of the elbow laterally, medially, and caudally using directly applied forces on the antebrachium while maintaining the humerus in a fixed position. During the attempt of luxation, the elbow was held in standing position at 110° of extension.^16^ An axial compression force was first applied, followed by valgus, varus or caudal forces to induce the ad hoc type of luxation.

## 3 RESULTS

A total of 10 cats (20 elbows) were used for the part I, and 5 (10 elbows) for part II.

### 3.1 Part I

No elbow luxation was observed by indirectly applied rotational forces.

Luxation was possible only by directly applied forces on the antebrachium. Lateral luxation was possible in all specimens (20/20 elbows) after transection of lateral collateral ligament and both parts of the medial collateral ligament. Medial luxation was not possible with transection of medial and lateral collateral ligaments (0/20 elbows). Caudal luxation was not possible with only collateral ligaments transection (0/20 elbows) but observed in all elbows after addition of olecranon ligament transection (20/20 elbows).

### 3.2 Part II

No elbow luxation was observed by indirectly applied rotational forces.

Luxation was possible only by directly applied forces on the antebrachium. Lateral luxation was observed after transection of olecranon ligament and both parts of medial collateral ligament in all specimens (5/5 elbows). Medial luxation was observed after transection of olecranon ligament and lateral collateral ligament in all specimens (5/5 elbows). Caudal luxation was observed after transection of olecranon ligament and lateral collateral ligament (10/10 elbows). Caudal luxation was not possible with transection of olecranon ligament and medial collateral ligament only.

Table 1 summarizes the results and identifies the minimum ligament transections required to induce each of the three types of luxation tested.

**TABLE 1:** Summary of the results and identification of the minimum ligament transections required to induce each of the three types of luxation tested.

	Lateral collateral ligament	Medial collateral ligament	Olecranon ligament
Lateral luxation	✓	✓	
		✓	✓
Medial luxation	✓		✓
Caudal luxation	✓		✓

## 4 DISCUSSION

**FIGURE 6:**
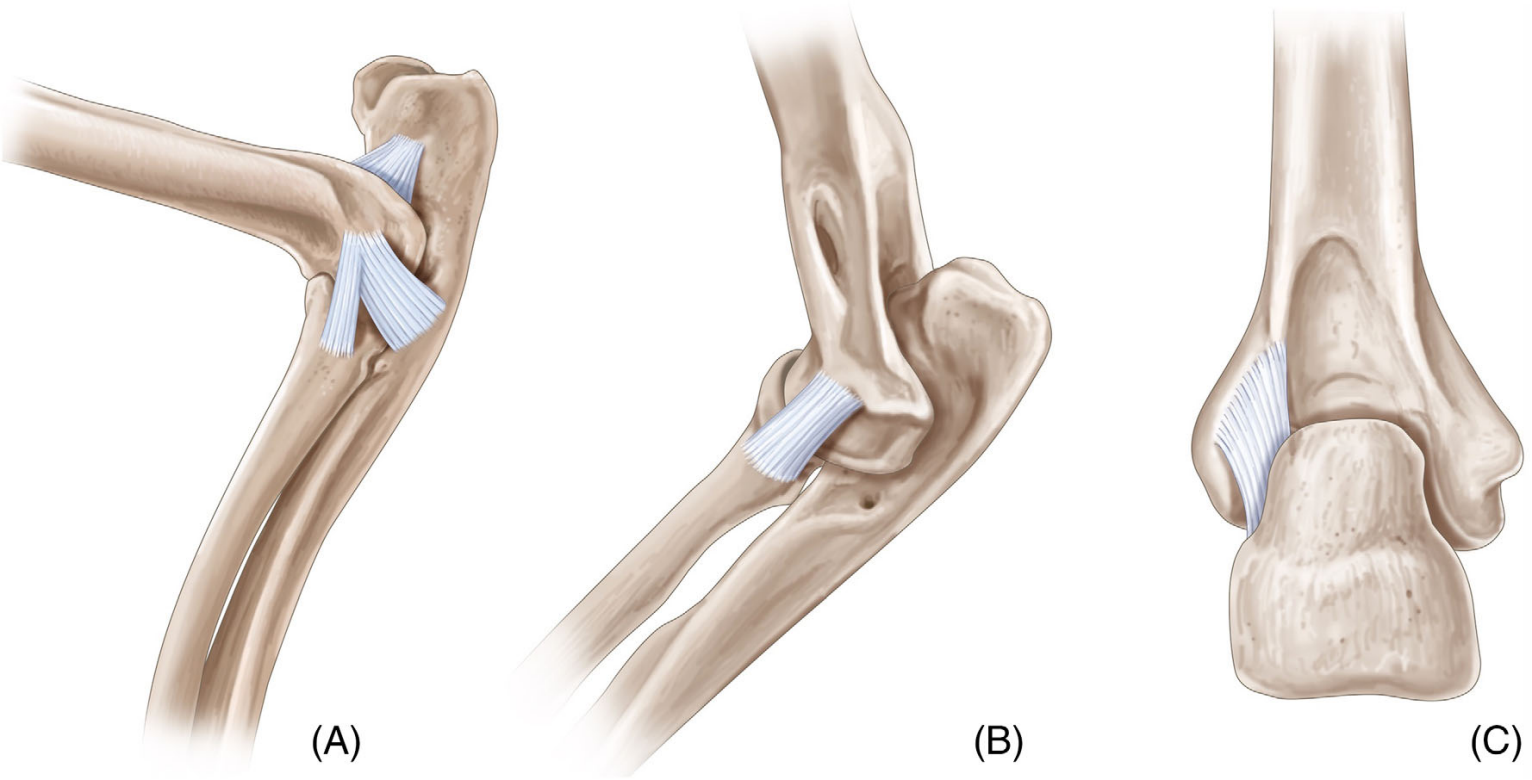
Illustration of collateral and olecranon ligaments in feline elbow. (A) Medial view, (B) lateral view and (C) caudoproximal view.

This study found that lateral elbow luxation is possible in cats after transection of medial and lateral collateral ligaments. Our study also highlights the major role of the olecranon ligament in elbow stability. Lateral elbow luxation is also possible after transection of olecranon and medial collateral ligaments. Medial elbow luxation is possible after transection of olecranon and lateral collateral ligaments. Caudal elbow luxation is possible with transection of olecranon and lateral collateral ligaments. Our findings provide further clarity on the anatomy of the collateral and olecranon ligaments in feline elbows (Figure 6). Contrary to some previous descriptions that suggest the LCL has both radial and ulnar attachments, our study confirmed that the LCL originated from the lateral epicondyle of the humerus and inserted exclusively on the radius, without any attachment to the ulna. Additionally, the LCL consisted of two distinct portions: a superficial part attaching to the proximal extremity of the radius and a deep part blending with the annular ligament, which encircles the head of the radius. Engelke and colleagues were the first team to reference the olecranon ligament to our knowledge.^18^, ^21^ The authors of this study were surprised to find that these studies are hardly ever cited in the veterinary literature, particularly in surgery despite the significance of these findings.

The Campbell's test can be used to determine collateral ligament integrity but has also been used in an extreme version to induce iatrogenic elbow luxation in the study of Farrell et al.^1^ In the latter, elbow luxation has been demonstrated only in two of six elbows by application of indirect rotational forces. In our study, no luxation was encountered by sole application of indirect rotational forces. The rotational forces applied were probably greater in Farrell's study, considering the reported fractures. High‐energy indirect rotational forces have been reported to be the cause of traumatic elbow luxation in dogs.^5^, ^6^ However, the most common causes of elbow luxation in dogs and cats are vehicular trauma and falls from heights.^3^, ^5^, ^6^ Similarly in humans, a fall on an outstretched hand or high‐impact trauma is the most common cause of elbow luxation.^24^, ^25^ Injuries have been reported to occur by a coupled 3‐dimensional rotatory motion pattern involving angulation around all three axes and displacement along ≥1 axes.^26^ O'Driscoll et al. described the mechanism of elbow posterolateral luxation during a fall onto an outstretched hand as a combination of axial compression, supination and valgus.^26^ This scenario could easily be translated to veterinary medicine where an axial force on the elbow is produced as the elbow flexes. With the body rotating on the paw, valgus/varus moments and rotation are applied to the elbow. However, different mechanisms may result in an elbow luxation with different patterns of soft tissue injuries.^25^ Nevertheless, it is highly unlikely that a single type of force, such as rotation alone, would be solely responsible for elbow luxation in dogs and cats. By applying direct forces to the proximal antebrachium—specifically axial, varus, valgus, and caudal forces—we were able to replicate the effect of two concurrent force vectors. Our results were repeatable in all the specimens tested. Application of forces was not quantitatively assessed. However, the tests were performed by the same operator in all specimens.

The cdMCL is considered stronger.^16^ In our testing protocol, transection of the cdMCL was consistently performed first to assess whether its isolated disruption could result in luxation. However, our findings indicate that rupture of both the cranial and caudal components of the MCL is necessary to induce elbow luxation. Voss et al. recommended reconstructing only the cdMCL when repairing MCL injuries in cats.^14^ Although we did not specifically assess the role of the crMCL by transecting it first, our findings suggest that its contribution is significant, as both components of the MCL must be transected to induce luxation. The transcondylar tunnels and biaxial suture repair described by Farrell et al. could be more appropriate because the two parts of the MCL are considered.^1^

Lateral elbow luxations account for approximately 65% of cases in cats.^6^ Medial and caudal luxations have also been documented in cats.^6^, ^14^, ^27^, ^28^ Our results indicate that the olecranon ligament plays a critical role in the pathophysiology of caudal elbow luxation in cats. Therefore, replicating the function of the olecranon ligament should be considered in the surgical management of caudal elbow luxation. The transcondylar tunnels and biaxial suture repair might be applicable for this type of luxation because of the transcondylar and transulnar tunnels.^1^ A tension is created between the ulna and the humerus, counteracting the cranial displacement of the humerus. A stabilization of the elbow in a flexed position with an external skeletal fixation for 3 weeks has been proposed for management of caudal elbow luxation after closed reduction in two cats.^27^ The authors found that the joint was more stable in flexion than in extension. This finding can be explained by the action and role of the olecranon ligament, which is put under tension when the elbow is flexed.

In contrast to the collateral ligaments, there is currently no test to assess the integrity of the olecranon ligament. Even though not investigated, a caudal drawer may be elicited (caudal motion of ulna/radius in relation to the humeral condyle). Further investigation is warranted to identify an appropriate test to assess the integrity of the olecranon ligament. Our results showed that elbow luxation (medial, lateral or caudal) can occur as a consequence of the rupture of one of the collateral ligaments and the olecranon ligament. After reduction of an elbow luxation and when assessing the integrity of the collateral ligaments by Campbell's test, if only one of the collateral ligaments appears severed, damage to the olecranon ligament should be considered.

The choice of a 110° elbow extension angle for luxation testing was guided by our objective to replicate in vivo conditions, and that this angle reflects the natural standing position in cats.^16^ However, just as it is unlikely that a single force vector is solely responsible for a luxation, it is also improbable that dislocation occurs at a fixed joint angle, given that axial loading continues to act as the elbow flexes from an extended or hyperextended position. Interestingly, in humans, 92% of elbow luxation occur at or near full extension.^28^ The authors cannot exclude the possibility that the results might differ at other angles of extension or flexion.

The limitations of the study include several factors that may impact the interpretation of the findings. First, the number of specimens tested, while significantly greater than in the study by Farrell et al.,^1^ may still not be sufficient to fully represent the variability found in a broader population. The lack of pretest radiographs prevented the identification of potential orthopedic conditions, such as medial epicondylitis, that could have influenced the results.^29^ However, no macroscopic abnormalities were observed during testing. This study did not measure the force required to induce an elbow luxation. Most biomechanical studies are either quasi‐static or cyclic tests; hence, they would not accurately replicate the dynamic forces encountered during traumatic events. The use of frozen cadavers may have altered the mechanical properties of the ligaments, potentially affecting the results. Finally, the study focused on isolated ligament injuries, but luxation injuries likely involve a complex interplay of factors, including ligaments, joint capsule, muscle insertions and origins, which contribute to the overall resistance to luxation.

## AUTHOR CONTRIBUTIONS

Hamon M, DVM, DECVS: Manuscript authorship, study design, data gathering, data assessment, drafting of the manuscript, revision of the manuscript, and approval of the final version of the submitted manuscript. Malreddy PR, BVSc AH, MS: Study design, performance of anatomic preliminary evaluation, approval of the final version of the submitted manuscript. Curtis AK, DVM: Study design, performance of anatomic preliminary evaluation, approval of the final version of the submitted manuscript. Picavet PP, DVM, MS, DECVS: Manuscript authorship, study design, data gathering, data assessment, drafting of the manuscript, revision of the manuscript, and approval of the final version of the submitted manuscript.

## CONFLICT OF INTEREST STATEMENT

The authors declare no conflict of interest.

## Data Availability

Data available on request from the authors.
